# Supplementary material for: Quantitative, Targeted Analysis of Gut Microbiota Derived Metabolites Provides Novel Biomarkers of Early Diabetic Kidney Disease in Type 2 Diabetes Mellitus Patients
Source: Biomolecules. 2023 Jul 7;13(7):1086. doi: 10.3390/biom13071086 (PMC10377254; doi:10.3390/biom13071086)
Supplement: Supplementary file 1 [file biomolecules-13-01086-s001.zip › biomolecules-2412821-supplementary.pdf]

Table S1A. Statistical data obtained by One way ANOVA including the Fidher LSD post-hoc test for the eight metabolites targeted in serum of controls *vs* P1-P3 subgroups: f-value, p-value, FDR and significance of differences according to post-hoc Fisher's LSD.

Molecule	f.value	p.value	FDR	Fisher's LSD
Arginine	11.907	0.0002	0.0001	C - P1; C - P2; C - P3; P1 - P2; P1 - P3; P3 - P2
Hippuric acid	6.718	0.0001	0.001	C - P1; C - P2; C - P3; P2 - P1; P1 - P3; P2 - P3
Sorbitol	6.179	0.001	0.002	C - P1; C - P2; P3 - C; P2 - P1; P3 - P1; P3 - P2
Dimethylarginine	2.281	0.083	0.167	P1 - C; P2 - C; C - P3; P2 - P1; P1 - P3; P2 - P3
Butenoyl carnitine	1.909	0.133	0.212	P1 - C; P2 - C; P3 - C; P1 - P2; P3 - P1; P3 - P2
L-Acetylcarnitine	1.750	0.161	0.215	P1 - C; C - P2; C - P3; P1 - P2; P1 - P3; P3 - P2
Creatinine (S)	1.497	0.220	0.251	P1 - C; C - P2; P3 - C; P1 - P2; P3 - P1; P3 - P2
Indoxyl sulfate	0.381	0.767	0.767	C - P1; P2 - C; P3 - C; P2 - P1; P3 - P1; P3 - P2

Table S1B. Statistical data obtained by One way ANOVA including the Fisher LSD post-hoc test for the eight metabolites targeted in urine of controls *vs* P1-P3 subgroups: f-value, p-value, FDR and significance of differences according to post-hoc Fisher's LSD.

	f.value	p.value	FDR	Fisher's LSD
p-Cresylsulfate	22.225	0.0001	0.0001	C - P1; C - P2; C - P3; P1 - P2; P1 - P3; P3 - P2
Arginine	7.872	0.0001	0.0002	C - P1; C - P2; C - P3; P1 - P2; P1 - P3; P2 - P3
Indoxyl sulfate	4.082	0.009	0.023	P1 - C; P2 - C; P3 - C; P2 - P1; P3 - P1; P3 - P2
Hippuric acid	1.958	0.125	0.250	C - P1; C - P2; C - P3; P2 - P1; P3 - P1; P2 - P3
L-Acetylcarnitine	1.581	0.198	0.317	C - P1; C - P2; C - P3; P2 - P1; P1 - P3; P2 - P3
Creatinine (U)	1.143	0.335	0.447	C - P1; P2 - C; P3 - C; P2 - P1; P3 - P1; P2 - P3
Butenoyl carnitine	0.987	0.402	0.459	C - P1; P2 - C; P3 - C; P2 - P1; P3 - P1; P3 - P2
Dimethylarginine	0.223	0.880	0.880	C - P1; C - P2; C - P3; P2 - P1; P3 - P1; P2 - P3

Figure S1 shows graphically the differences (expressed in MS peak intensities) between the groups C, P1, P2 and P3, for each potential serum biomarker targeted in blood serum, considering their original concentrations and the normalized ones by median values.

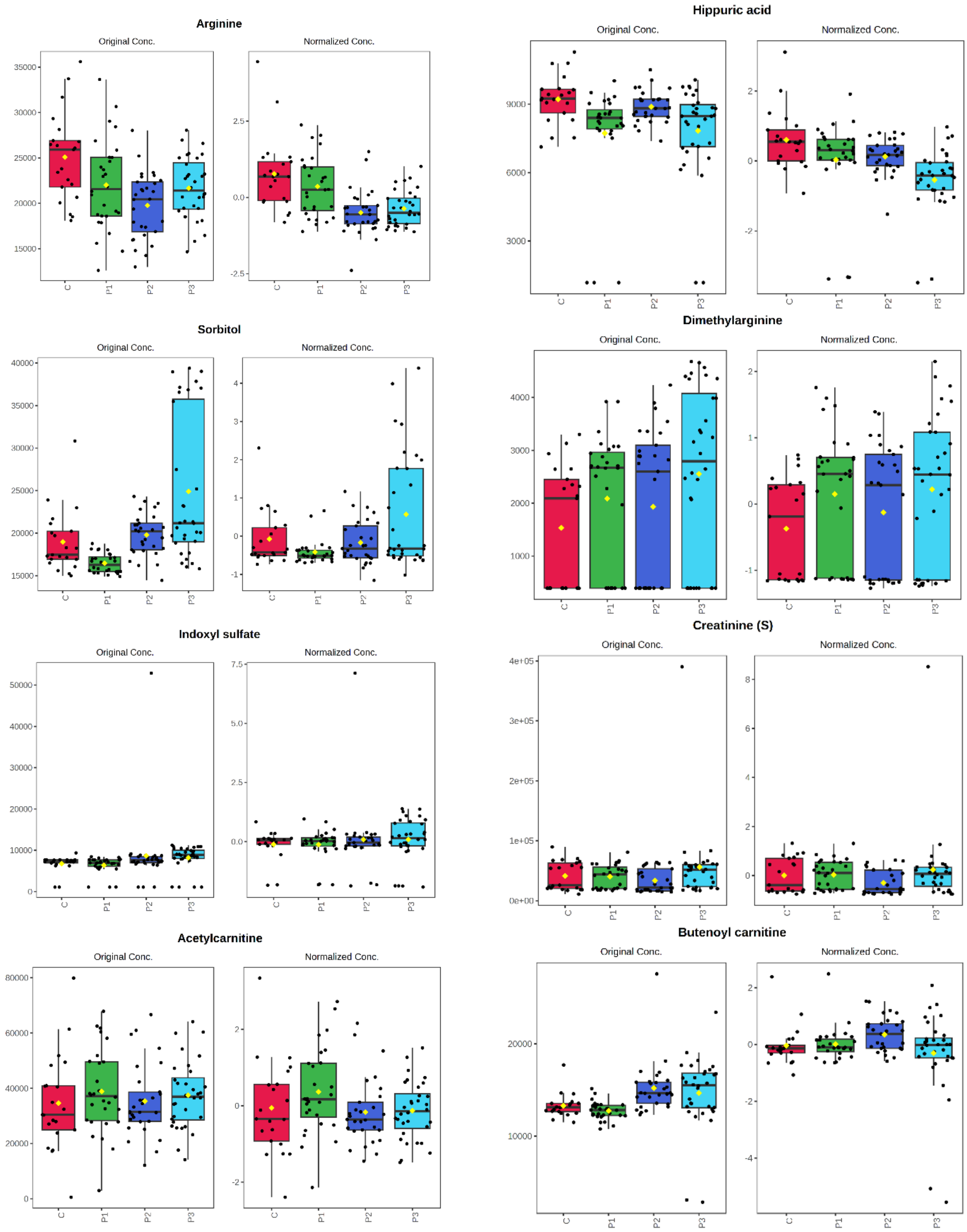

Figure S1. Graphic representation of the differences (expressed in MS peak intensities) between the groups C, P1, P2 and P3, for each of the eight serum potential biomarkers. The original and normalized (sample normalization by median values) are presented.

Figure S2 shows graphically the differences (expressed in MS peak intensities) between the groups C, P1, P2 and P3, for each potential targeted biomarker in urine considering their original concentrations and the normalized ones by median values.

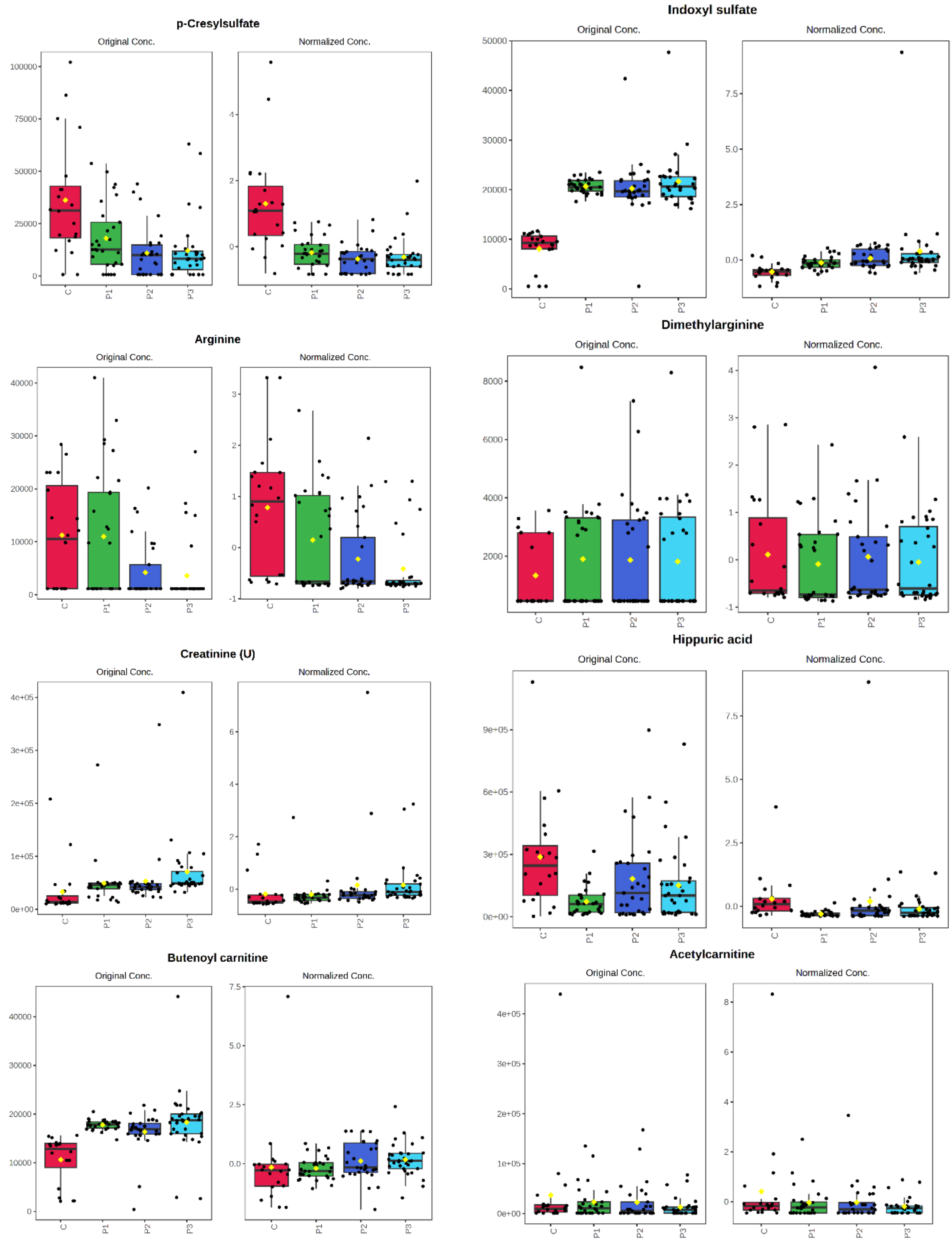

Figure S2. Graphic representation of the differences (expressed in MS peak intensities) between the groups C, P1, P2 and P3, for each of the eight urine potential biomarkers. The original and normalized (sample normalization by median values) are presented.

Calibration curves for the targeted metabolites

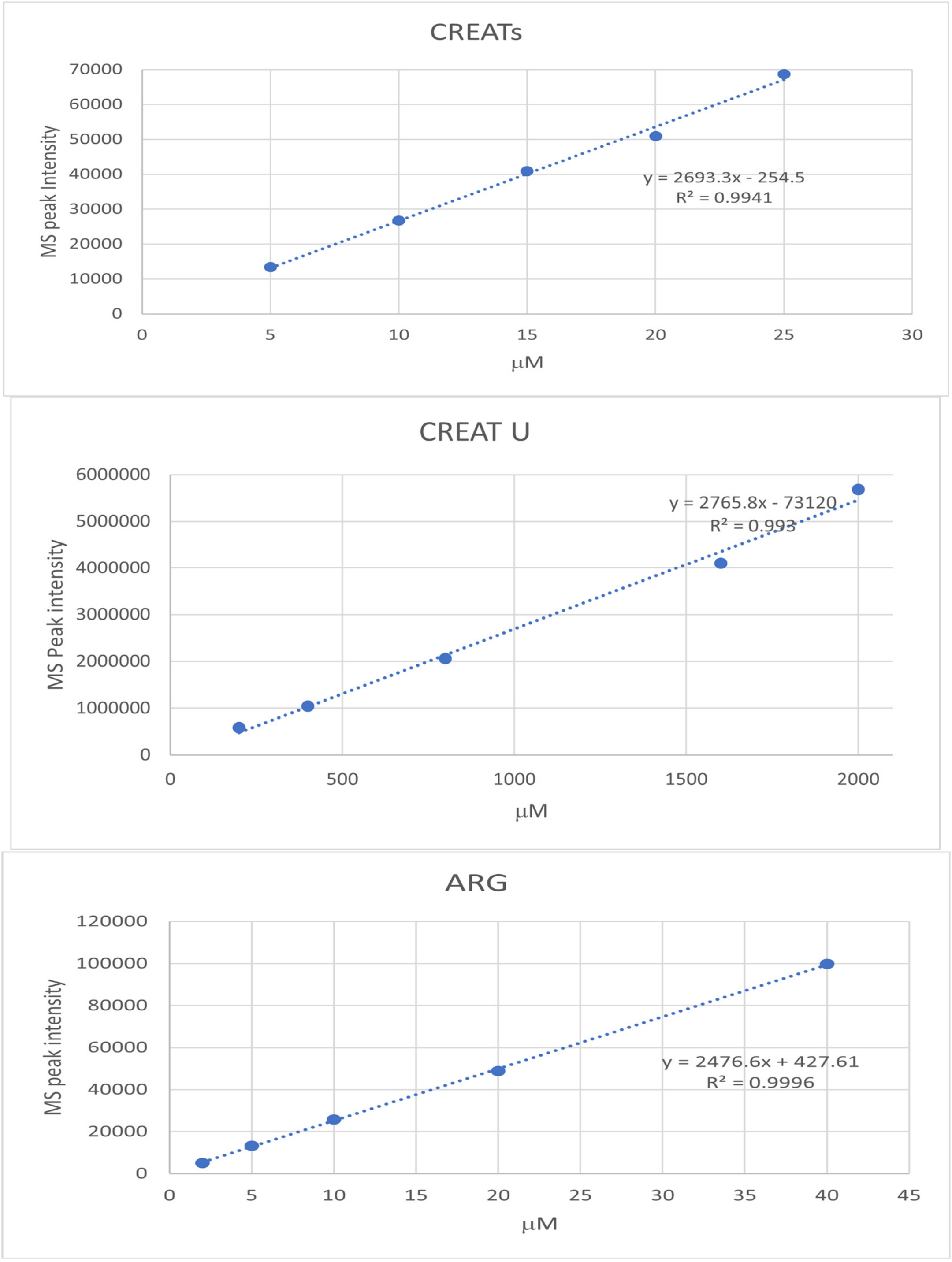

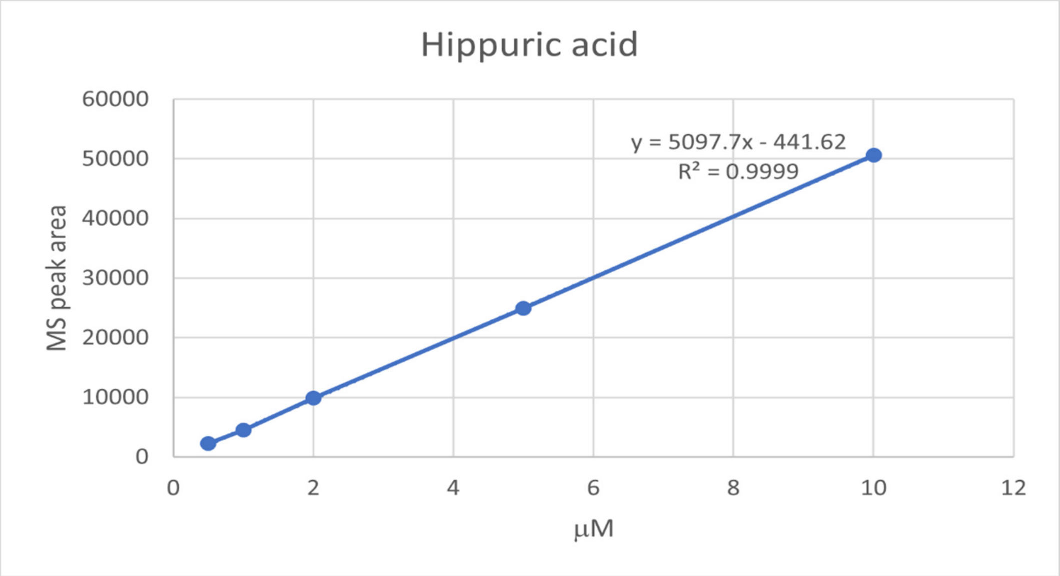

Hippuric acid
y = 5097.7x - 441.62
R² = 0.9999
MS peak area
60000
50000
40000
30000
20000
10000
0
0
2
4
6
8
10
12
µM

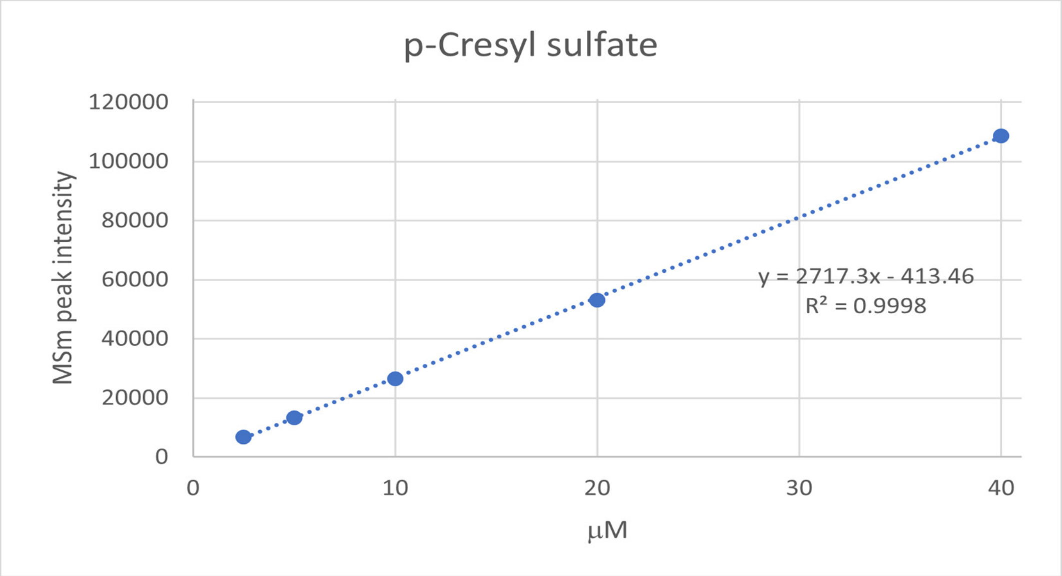

p-Cresyl sulfate
y = 2717.3x - 413.46
R² = 0.9998
MSm peak intensity
120000
100000
80000
60000
40000
20000
0
0
10
20
30
40
µM

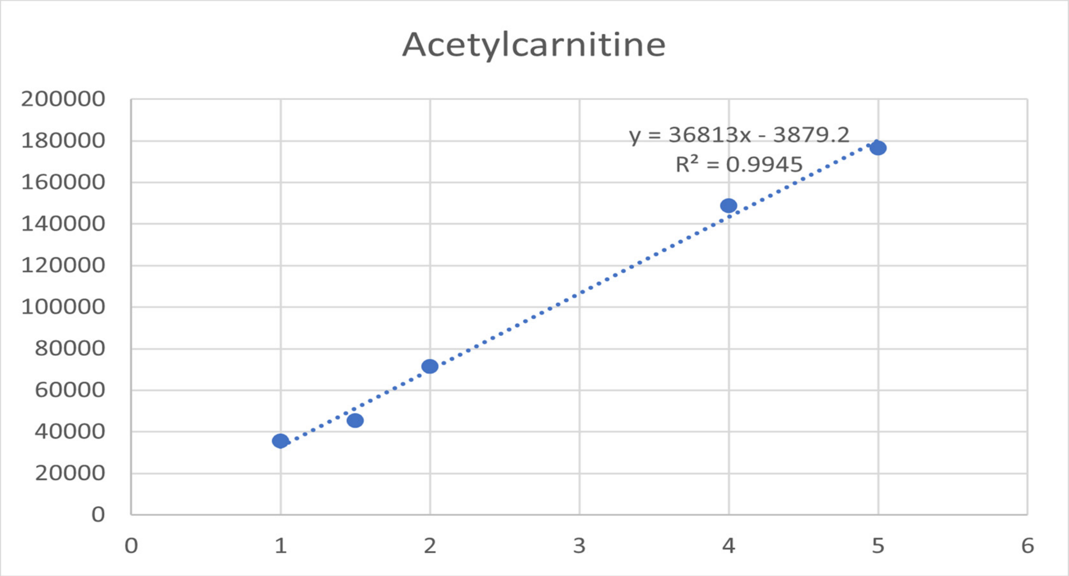

Acetylcarnitine
y = 36813x - 3879.2
R² = 0.9945
200000
180000
160000
140000
120000
100000
80000
60000
40000
20000
0
0
1
2
3
4
5
6

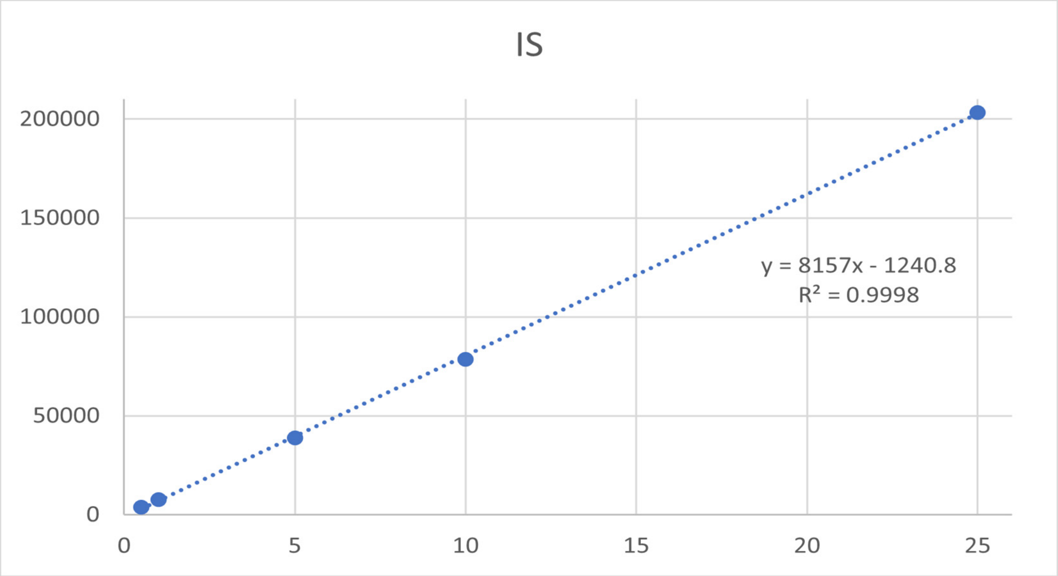
IS
200000
150000
100000
50000
0
0
5
10
15
20
25
y = 8157x - 1240.8
R² = 0.9998

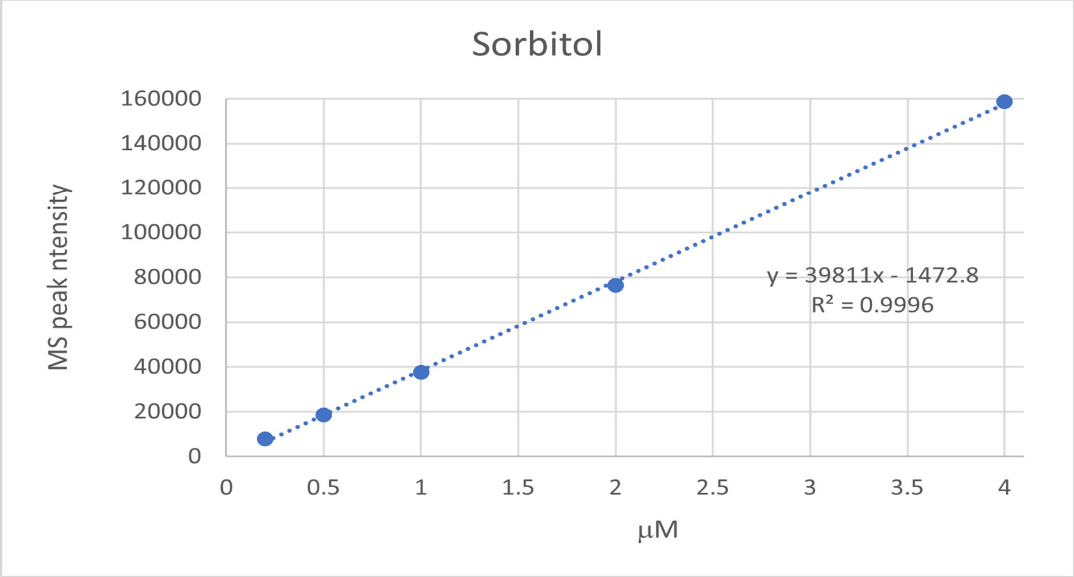
Sorbitol
MS peak ntensity
160000
140000
120000
100000
80000
60000
40000
20000
0
0
0.5
1
1.5
2
2.5
3
3.5
4
μM
y = 39811x - 1472.8
R² = 0.9996

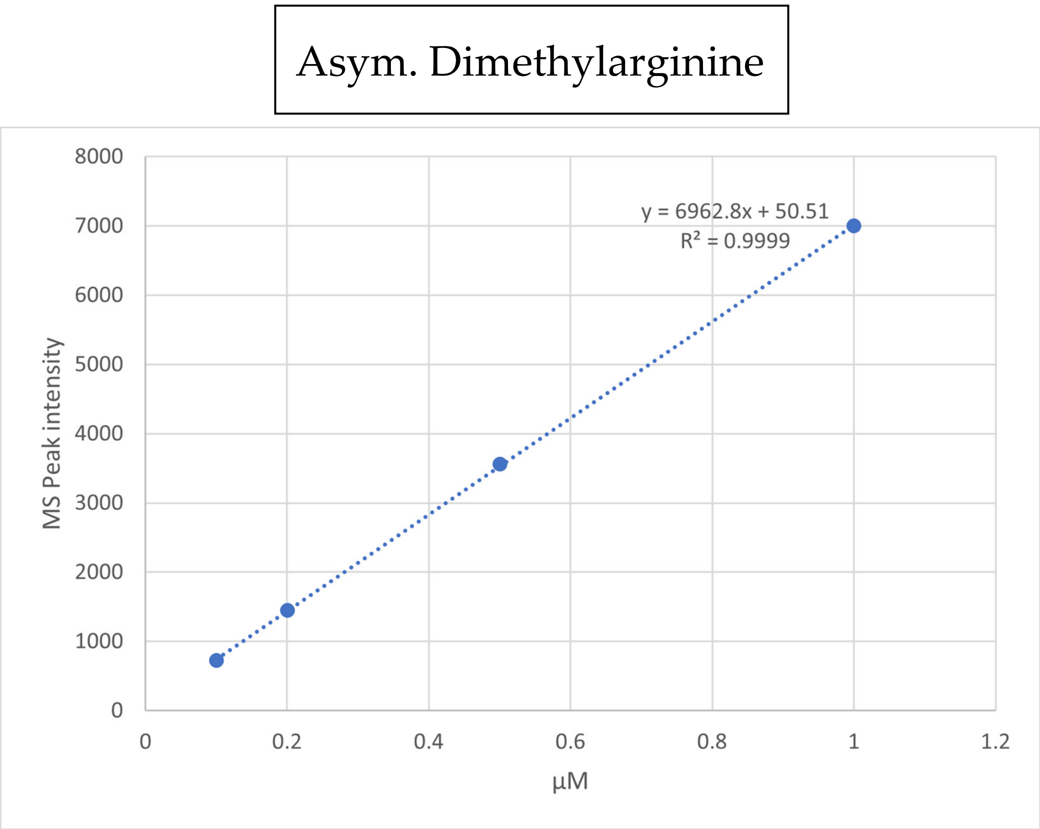

Figure S3. Calibration curves for the selected putative biomarkers: creatinine(from blood serum and urine), Arginine, Hippuric acid, p-Cresyl sulfate, Acetyl carnitine, Indoxyl sulfate (IS), sorbitol, Asymmetric Dimethylarginine.

Table S2. Untargeted and targeted analyses of metabolites in serum and urine based on mean DKD/mean C ratios.

		Untargeted (PI) - mean DKD/mean C ratio	Targeted (µM) mean DKD/mean C ratio
**Serum**	**Identification**		
175.1306	Arginine	0.80	0.80
180.1716	Hippuric acid	0.90	0.90
183.0940	Sorbitol	0.99	0.99
204.1369	L-Acetylcarnitine	1.01	1.01
214.2676	Indoxyl sulfate	1.21	1.21
230.2668	Butenoyl carnitine	1.08	1.07
**Urine**			
175.1306	Arginine	1.06	1.06
180.1716	Hippuric acid	1.10	1.10
204.1369	L-Acetylcarnitine	2.25	1.51
214.2676	Indoxyl sulfate	3.02	2.65
189.1594	p-Cresylsulfate	1.91	1.87
230.2668	Butenoyl carnitine	3.50	2
